# Treatment of alcohol dependence in patients with co-morbid major depressive disorder – predictors for the outcomes with memantine and escitalopram medication

**DOI:** 10.1186/1747-597X-3-20

**Published:** 2008-10-03

**Authors:** Leea H Muhonen, Jari Lahti, David Sinclair, Jouko Lönnqvist, Hannu Alho

**Affiliations:** 1 National Public Health Institute, Department of Mental Health and Alcohol Research, Finland; 2 Department of Psychiatry, University of Helsinki, Finland; 3 Helsinki University Central Hospital, Finland; 4 Research Unit of Substance Abuse Medicine, University of Helsinki and Helsinki University Central Hospital, Finland; 5 Department of Psychology, University of Helsinki, Finland; 6 Helsinki City Health Care Center, Finland

## Abstract

**Background:**

Alcohol dependence comorbid with major depressive disorder poses a major challenge in the clinical setting. The results in the treatment with selective serotonin re-uptake inhibitors have been conflicting. Thus, we compared in alcohol-dependent patients with co-morbid major depressive disorder the selective serotonin re-uptake inhibitor escitalopram to a compound that acts on different transporter system and may reduce craving, the glutamate receptor antagonist memantine.

**Methods:**

Eighty alcohol-dependent patients comorbid with major depressive disorder in municipal alcohol clinics were randomized 1:1 to receive memantine 20 mg or escitalopram 20 mg in a double-blind manner. During the 26-week study period patients continued their routine treatment at the clinics. Abstinence was not required but encouraged. The patients attended visits weekly during the first month, and then at 3 and at 6 months. Outcome measures were Alcohol Use Disorders Identification Test (AUDIT), Obsessive Compulsive Drinking Scale (OCDS) and Drinking Diary.

**Results:**

The completion rate was high in both groups, especially among the patients who had been abstinent at the beginning of the study. However, among those patients who were not abstinent at baseline, 47% in both groups discontinued the study. Numbers of abstinent days were high in both groups throughout the study. Alcohol consumption measured by the AUDIT QF (quantity-frequency) score was significantly reduced in both groups, as was the craving for alcohol measured by the OCDS. Early age at first alcohol intoxication predicted poor treatment outcomes in patients treated with escitalopram, and the same was seen with the early onset of the first depressive episode. The same predictive effects were not found in patients treated with memantine.

**Conclusion:**

Our results indicate that both memantine and escitalopram are useful adjunct medications for the treatment of alcohol dependence co-morbid with major depression. Memantine was at least as effective with regard to drinking as escitalopram. We believe that a direct comparison of memantine, with the commonly used escitalopram, can provide useful information for clinicians on the treatment of alcohol dependency co-morbid with MDD.

**Trial registration:**

ClinicalTrials.gov Identifier # NCT00368862

## Background

The lifetime prevalence of alcohol dependence as well as co-morbidity with depressive disorders is high. For example, the lifetime prevalence of alcoholism was 5.4% in the United States among individuals over the age of 18 years according to the National Comorbidity Survey Replication (NCS-R)[1]. Among these alcohol-dependent patients, co-morbidity with depressive disorders was 24.3% in men and 48.5% in women [2]. Alcohol-dependent patients who are co-morbid for major depressive disorder (MDD) constitute a patient group whose pharmaceutical treatment has been particularly difficult [3].

It has been proposed that SSRIs are most beneficial for the treatment of alcohol dependence [17,18]. However, the results are inconsistent. Some studies supported the efficacy of zimilidine and citalopram in alcoholics [18-20]. In alcoholism without depression, SSRIs have shown positive results in cases of less severe drinking [5,21]. In some studies SSRIs were found even to be worse than placebo [22,23] especially when treating early onset (Type B, Type II) subtypes of alcoholics [24,25]. Treatment with SSRIs for alcohol dependence with co-morbid major depressive disorder (MDD), however, has generally produced positive results [11,12,26]. Escitalopram, the S-enantiomer of citalopram, is the most selective of the SSRI antidepressants [27]. It is now widely used for the treatment of depression [20]. In a recent review escitalopram was found to have the highest efficacy among antidepressants in the treatment of severe depression [28] but there are no studies on escitalopram in the treatment of MDD comorbid with alcohol dependence.

Acamprosate is a weak NMDA modulator which acts as an antagonist at the mGluR5 metabotropic glutamate receptor [29]. Acamprosate is approved in many countries for the treatment of alcohol dependence [30] although some recent studies [31,32] have not found significant benefits.

Memantine is a non-competitive ionotropic NMDA receptor blocker. It is FDA approved for the treatment of moderate to severe Alzheimer's disease [33]. Memantine has been shown to block ethanol-induced up-regulation of NMDA receptors [34]. Rat studies have shown that memantine may reduce alcohol craving [35-39]. Recent clinical studies show that memantine may suppresses the craving for alcohol in moderate drinkers when deprived, but not when drinking [40]. In recovering alcohol-dependent patients memantine seemed to reduce craving [41], while in actively drinking alcohol-dependent non-depressive patients, memantine did not reduce craving or alcohol consumption [42]. The studies of memantine in the treatment of depression are rare: when treated therapy-resistant depressive patients, Zarate did not find any recovering [43], while in our recent study, memantine was comparable with escitalopram in the treatment of major depression comorbid with alcohol dependence [44]. We hypothesized that the NMDA receptor antagonist memantine may reduce also craving and alcohol consumption in depressive alcoholics. The aim of this study was to compare effects of NMDA receptor antagonist memantine to escitalopram on alcohol consumption, in a natural sample of treatment-seeking alcohol-dependent patients (both actively drinking and recovering) with comorbid MDD. The possible predictors for treatment outcomes were studied also.

## Methods

### Study participants

Men and women aged 26 to 65 years who were voluntarily seeking outpatient treatment for alcohol problems at three Helsinki municipal Alcohol-clinics (A-clinics). Patients with a history of heavy drinking (averaging five or more daily drinks for men and four or more daily drinks for women) for at least ten years, significant depression defined by the Beck Depression Inventory II (BDI-II > 16), and who were interested in voluntarily taking part in the study were recommended by their A-clinic doctor or social worker therapist to be interviewed and screened by the study physician. The patients were interviewed by the study doctor (psychiatrist LM) applying the Structured Clinical Interview for DSM- IV (SCID) and were required to meet the criteria of both alcohol dependence and MDD according to DSM-IV-TR. Abstinence was not required but encouraged. The time since the last prior inpatient detoxification had to be at least four weeks. In addition, the eligible patients had to be currently in a depressive episode lasting for more than two weeks. The exclusion criteria included other substance use dependence screened by urine test (amphetamine, benzodiazepines, cocaine, tetrahydrocannabinol and opiates, schizophrenia or other psychotic disorder, and bipolar I and II disorder, acute risk of suicide, pregnancy or breastfeeding, a severe untreated somatic problem, or a serious dysfunction of the liver (aspartate aminotransferase [AST] and alanine aminotransferase [ALT] > 200), and mental disability. Other medications prescribed by participants' physicians were allowed, with the exception of other antidepressants. All patients were Caucasian, and 55% were men. There were no significant differences between the groups in either their demographic characteristics or their initial alcohol and depressive measures [44]. The mean length of the present depressive period was 35 months. Current alcohol use was reported by 17 patients (43.6%) in the memantine group and 17 (42.5%) in the escitalopram group. The number of A-clinic visits (psychosocial counseling) during the study period was similar: in the memantine group 7.7 ± 8.8 (mean ± SD) and in the escitalopram group 7.1 ± 9.2. [44].

All 58 subjects who completed the study attended all appointments and showed at least 80% compliance based on tablet counts. The average consumption (mg) of medication did not differ between the two medication groups: during the first 12 weeks, for memantine 17.4 ± 0.5 mg and for escitalopram 16.9 ± 0.6 mg; and during weeks 13–26, for memantine 17.4 ± 0.6 mg and for escitalopram 15.9 ± 0.8 mg.

### Ethics

The study was approved by the independent Hospital District of Helsinki and Uusimaa, Ethical Committee (permission 22/2004) and the Finnish National Agency of Medicine (KL# 87/2004). The study was conducted according the ICH Guidelines for Good Clinical Practice and the 1964 Declaration of Helsinki. The study was registered on the National Public Health study registry in March, 2005 (172–9), and the ClinicalTrials.gov Identifier (trial # NCT00368862). All patients had to be able to read and understand the patient information sheet and sign the informed consent. All participants were free to stop the study medication whenever they wanted. The patients were not paid or otherwise reimbursed for participation.

### Study design

Eighty-nine patients were initially screened. A screening interview (SCID) was conducted to confirm the diagnoses of MDD and alcohol dependence. Patients completed questionnaires including the Obsessive-Compulsive Drinking Scale (OCDS [45]) and the Alcohol Use Disorders Identification Test (AUDIT) [46]. AUDIT-QF [47], and AUDIT-3 [48] were used for a detailed drinking analysis. The recording of alcohol consumption during the 26-week treatment period was done with a personal drinking diary for all days, including abstinent days [49].

All patients meeting the inclusion criteria were randomly assigned to the memantine or escitalopram group using a 1:1 ratio (n = 40 + 40). Eligible patients received orally either 20 mg/day escitalopram or 20 mg/day memantine. The starting dose was 5 mg/day for both drugs and was increased at weekly intervals by 5 mg/day to 20 mg/day. Patients were instructed to take the study medication in the morning. Patients were permitted to telephone the study physician at any time. If the patient did not appear at a scheduled visit, a new appointment was offered.

During the 26-week treatment period, the patients returned to the study site at weeks 1, 2, 4, 12 ± 2, and 26 ± 2 for data collection and for medication checking and dispensing. At each visit, the drinking diary and the study medication intake since the previous visit were recorded from the medication diary. The study medication was ensured by pill count from the returned blister-packs. Outcomes were recorded on specific weeks: OCDS (weeks 0, 4, 12 and 26); AUDIT (week 0, 12 and 26, the later ones modified to report the events in the previous month). Clinical laboratory tests (MCV, AST, ALT, CDT, and GGT) were taken at the beginning of the study and were repeated at weeks 4, 12, and 26, to ensure the safety of the medication. No breath or blood test for alcohol was performed, but if the patient was obviously intoxicated, a new appointment was offered. The study was monitored by an independent organization, Medikalla Oy, Medfiles, Turku.

### Statistical analysis

All primary and secondary outcome statistical analysis was performed by an independent source (Medikalla Oy, MedFiles, Turku). All statistical evaluation utilized SAS Procedures in SAS^® ^system for Windows (Version 8.2), SAS-institute, Espoo, Finland.

Intent-to-treat sample, which included all randomized patients including two patients who discontinued early in the study and reported, taking no medication, were used in all tables and analyses. Descriptive statistics were calculated for all variables. Categorical variables were presented in frequencies tables (number of cases and percentages) by treatment. The numerical variables were tabulated by treatment. Baseline measures were analyzed by logistics regression or analysis of variance. All repeated dependent measures (drinking diary, OCDS, AUDIT, laboratory tests), were analyzed with analysis of variance for repeated measures (ANOVA) when treatment, time, and treatment * time interaction were in the model (PROC MIXED in SAS^®^) and responses to the specific question (Has your alcohol use diminished during the study?) were analyzed by logistic regression (PROG LOGOSTIC in SAS^®^).

Furthermore, predictors of treatment response by medication were analyzed with multiple linear regression analyses by adjusting for the baseline OCDS and AUDIT scores.

## Results

The drop out ratio was similar in the two groups: 11 out of 40 patients (27.5%) discontinued the study before the end of the 26-week period in both the memantine and the escitalopram groups. Two patients in both treatment groups (6.9%) used disulfiram, and one patient in both groups (3.4%) used a mood stabilizer.

### Alcohol consumption

**Figure 1:**
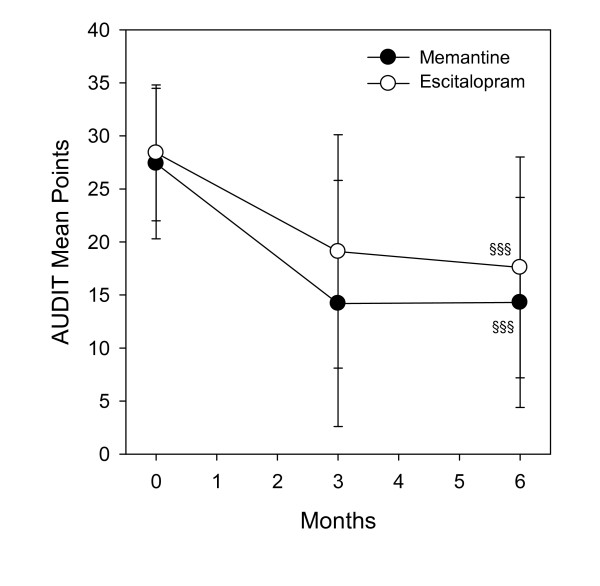
**Change in alcohol use measured by the AUDIT scores.** §§§Significant reduction from base values prior to treatment, p < .0001, ± SD.

The baseline AUDIT and alcohol use histories are similar in both groups (Table 1). AUDIT scores decreased (Fig. 1) from baseline in both groups, from 27.4 ± 7.1 to 14.3 ± 9.9 in the memantine group and from 28.4 ± 6.4 to 17.6 ± 10.4 in the escitalopram group. The overall reduction was highly significant (*F *[2.77] = 48.42, *p *< .0001) in both groups combined. The treatment by time interaction was not significant (*F *[2.77] = 1.19, *p *= 0.31).

**Table 1:** Demographic backgrounds.

**Variable**	**Memantine (n = 40)**	**Escitalopram (n = 40)**
Age (years, mean ± SD)	47.5(± 8.3)	47.9(± 8.3)
Gender, male (n, %)	23 (57.5)	21 (52.5)
First alcohol intoxication, (age, mean ± SD)	15.3 (± 3.8)	15.4 (± 2.3)
Onset of regular use of alcohol (age, mean ± SD)	20.7(± 6.7)	20.5 (± 6.3)
Onset of alcohol abuse (age mean ± SD)	29.5(± 8.1)	28.3(± 8.3)
Onset of alcohol dependence (age, mean ± SD)	30.6 (± 8.3)	29.1(± 8.5)
Audit baseline (mean ± SD)	27.4 (± 1.1)	28.4 (± 1.0)
No abstinence before study initiation (n, %)	17 (43.6)	17 (42.5)*
Alcohol problems among relatives (n, %),	31 (79.5)*	30 (76.9)*
Montgomery-Asberg depression rating scale (MADRS) baseline scores	25.8(± 4.4)	26.8 (± 4.1)
First depressive episode (age, mean ± SD)	27.8(± 12.3)	24.2 (± 13.0)
Total number of depressive episodes (mean ± SD)	10.0(± 7.1)	9.6(± 9.0)

There were no significant differences between the groups on any of the baseline socio-demographic background measures.

*missing information in one patient

Alcohol consumption measured by the AUDIT QF (quantity-frequency) score was significantly reduced in both groups: in the memantine group from 6.2 ± 1.7 to 4.1 ± 2.5 and from 6.1 ± 1.7 to 4.3 ± 2.3 in the escitalopram group (*F *[2.77] = 23.53, *p *< .0001). The treatment by time interaction was not significant (*F *[2.77] = 1.58, *p *= 0.21). The number of heavy drinking days measured by the AUDIT-3 score was also diminished significantly in both groups: for the memantine group from 2.9 ± 1.1 to 1.8 ± 1.3 and from 3.1 ± 1.0 to 2.4 ± 1.3 for the escitalopram group (*F *[2.77] = 20.29, *p *> .0001). The treatment by time interaction was not significant (*F *[2.77] = 1.37, *p *= 0.27).

**Figure 2:**
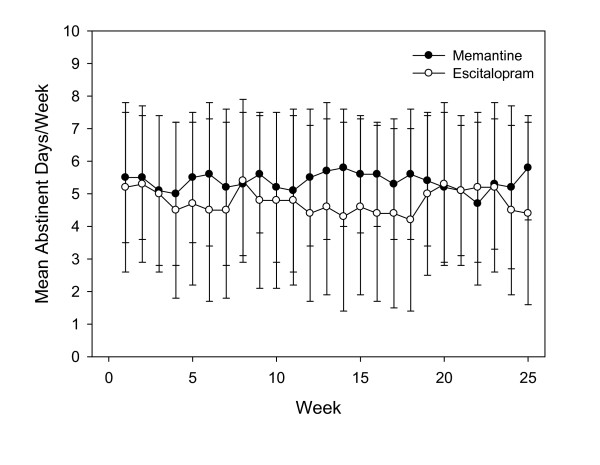
**Mean number of abstinent days per week.** No statistically significant difference between the treatment groups in mean number of days ± SD.

The number of abstinent days per week was high for both groups throughout the study. The treatment by time interaction in the number of abstinent days per week was not significant (*F *[2.74] = 0.07, *p *= 0.93) (Figure 2). The mean alcohol intake including abstinent days was 15.0 ± 2.6 g per day for the memantine group and 21.1 ± 3.6 g per day for the patients on escitalopram, with no significant difference between the groups (*F *[1.74] = 1.94, *p *= 0.17).

When questioned at the end of the intervention, 68.9% of the patients in the memantine group reported their alcohol use had decreased while 62.1% of the patients in the escitalopram group reported a decrease.

### Indicators of craving for alcohol

**Figure 3:**
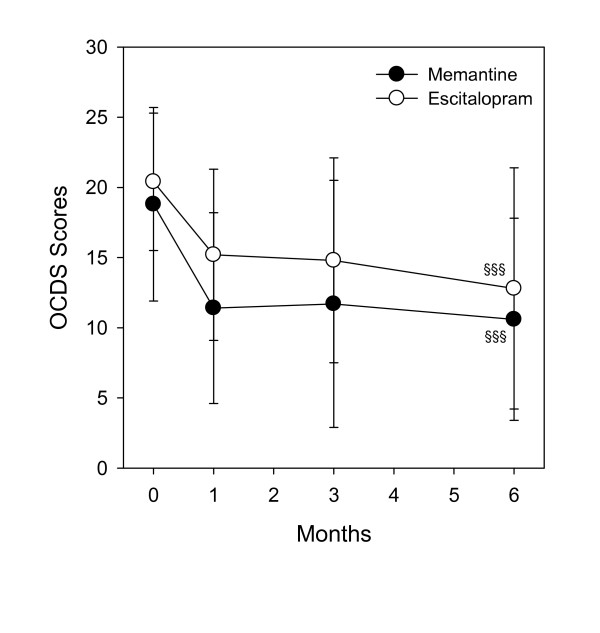
**Change in alcohol graving measured by the OCDS scores.** §§§significant reduction from base values prior to treatment, p < .0001, ± SD.

The OCDS total scores (Fig. 3) decreased in the memantine group from 18.8 ± 6.9 to 10.6 ± 7.2 and in the escitalopram group from 20.4 ± 4.9 to 12.8 ± 8.6. The overall reduction was highly significant (*F *[3.77] = 25.76, *p *< .0001) in both groups combined. The treatment by time interaction in the OCDS was not significant (*F *[3.77] = 0.69; *p *= 0.56).

In both groups, the mean serum concentrations of AST, ALT, GGT, and CDT were within normal limits, and there were no significant changes during the treatment period or any significant differences between the groups. The adverse events during the study are published in our previous article [44]. There was no significant difference in reporting adverse events between the medication groups.

### Predictors of the treatment outcomes

In general, those patients who were abstinent at the beginning of treatment were more likely to complete the treatment than those who were still drinking at the beginning (*χ*^2 ^= 6.51, *df *= 1, *p *= 0.011). This relationship was highly significant in the patients treated with memantine (*χ*^2 ^= 7.25, *df *= 1, *p *= 0.007): 8 of the 11 who dropped out were among the 17 (47.1%) who were active drinkers at the baseline. The relationship was in the same direction in the escitalopram group but failed to reach statistical significance (*χ*^2 ^= 0.901, *df *= 1, *p *= 0.343).

We tested by multiple linear regression analyses whether age at onset of depression and age at first alcohol intoxication predicted change during the six month treatment in the OCDS and the AUDIT scores. Age at onset of depression predicted differently change in the OCDS in the escitalopram group compared with the memantine group (*R*^2 ^change for interaction term = 0.05; *p *for treatment by age at onset of depression interaction = 0.05). In the escitalopram group, earlier onset of depression predicted less change in the OCDS scores during the six months (*B *= -0.31, 95% CI = -0.53 to -0.09, *p *= 0.008) whereas in the memantine group no such association existed (*B *= -0.02, 95% CI = -0.22 to 0.17, *p *= 0.81). There were no differences between the medication groups in the association between age at onset of depression and change in the AUDIT (*R*^2 ^change for interaction term = 0.02; *p *for interaction = 0.21) or in the association between age at first alcohol intoxication and change in the OCDS and the AUDIT (*R*^2 ^change for interaction term < 0.022; *p*-values for interactions > 0.21). Neither did age at onset of depression predict change in the AUDIT (*B *= -0.16, 95% CI = -0.37 to 0.05, *p *= 0.12) or age at first alcohol intoxication predict change in the OCDS (*B *= -0.68, 95% CI = -1.41 to 0.05, *p *= 0.07) and the AUDIT (*B *= -0.24, 95% CI = -1.62 to 1.13, *p *= 0.72) in the subjects when both treatment groups were combined.

## Discussion

Citalopram and escitalopram have been used in the treatment of alcohol dependence especially when co-morbid with major depressive disorder. Treatment outcomes with other SSRIs have not been consistent [3,15,26,50-53]. In our study, both memantine and escitalopram patients reported reduced alcohol craving and consumption, and patient compliance was good. However, the two study groups did not differ in alcohol craving, obsessive thoughts of drinking, compulsive drinking, alcohol consumption, maintaining abstinence, and number of abstinent days per week. Our study corroborates a recent study by Krupitsky et al. [41], who reported that memantine reduced alcohol cue-induced craving in recovering alcoholics.

Few possible predictive elements for the treatment of alcohol dependence comorbid with major depressive disorder with either escitalopram or memantine were observed. The abstinence at the beginning of the treatment predicted more likely the continuing of the treatment, especially in the memantine group. Evans et al. [42] found no effect of memantine in patients who were actively drinking at the beginning of treatment, which may explain our findings that such patients had a higher dropout rate than those who were abstinent at the start of treatment. Other predictor observed was the early age at onset of the first depressive episode, which leads to poor treatment outcome with escitalopram but not with memantine. However, due to multiple testing without a priori hypothesis, this difference should be interpreted with caution.

Our study has several limitations. It is a comparative study of two medications and is limited by the absence of a placebo group. Both treatment groups improved significantly and a placebo-effect could be significant in both groups, as has been observed in earlier studies [52]. Spontaneous recovery and intermittent periods of lower alcohol intake are of typical in alcohol dependence [54,55]. Therefore, we cannot determine whether the overall improvement in the present study was due to the medications, and our interpretations are limited to comparisons between memantine and escitalopram. Detoxification and a certain period of abstinence could influence the results.

Another limitation is the rather small number of patients, which may have been too low to detect a significant difference between the treatments. Socio-demographic indicators correspond well to those generally found among patients treated at Finnish A-clinics [56], suggesting that the present material represents a relatively unbiased sample. The only difference observed was the higher percentage of women, which can probably be attributed to the inclusion criterion of major depression. We did not attempt to distinguish between patients with primary depressive disorders and substance-induced depression. This situation corresponds to that at the onset of treatment; it is when the clinician has to decide which medications to prescribe. Our patients were treatment-seeking, so the option of providing no active medication was not accepted by either the treating professionals or the patients.

Our finding may suggest that memantine could be useful treatment for type one alcoholics (early onset) comorbid with depression. The finding that the early onset of the first depressive episode is a negative predictor for escitalopram treatment in alcohol dependence confirms our previous finding on treatment of this comorbidity regarding major depressive disorder in patients with this dual diagnosis [57].

It may be concluded that memantine seems comparable to escitalopram and could be used as well as escitalopram in the treatment of alcohol dependence comorbid with major depression. The results, therefore, warrant further studies of memantine in patients with alcohol dependence comorbid with major depression as well as of predictive signs of treatment outcome. However, because of the small number of study patients and the conventional statistics, the results should be taken with caution.

## Competing interests

The authors declare that they have no competing interests.

## Authors' contributions

LM, JLÖ and HA have made substantial contributions to conception and design, LM and HA for acquisition of data, LM, JLA, DJ and HA analysis and interpretation of data; all authors have been involved in drafting the manuscript or revising it critically for important intellectual content; and all authors have given final approval of the version to be published.
